# *N*-methyl-D-Aspartate Receptors Contribute to Complex Spike Signaling in Cerebellar Purkinje Cells: An *In vivo* Study in Mice

**DOI:** 10.3389/fncel.2016.00172

**Published:** 2016-06-30

**Authors:** Heng Liu, Yan Lan, Yan-Hua Bing, Chun-Ping Chu, De-Lai Qiu

**Affiliations:** ^1^Cellular Function Research Center, Yanbian UniversityYanji, China; ^2^Department of Physiology and Pathophysiology, College of Medicine, Yanbian UniversityYanji, China; ^3^Key Laboratory of Natural Resource of the Changbai Mountain and Functional Molecular of the Ministry of Education, Yanbian UniversityYanji, China

**Keywords:** *N*-methyl-D-aspartate (NMDA), cerebellar purkinje cell, *in vivo* whole-cell patch-clamp recording, complex spike (CS), after-hyperpolarization (AHP)

## Abstract

*N*-methyl-D-aspartate receptors (NMDARs) are post-synaptically expressed at climbing fiber-Purkinje cell (CF-PC) synapses in cerebellar cortex in adult mice and contributed to CF-PC synaptic transmission under *in vitro* conditions. In this study, we investigated the role of NMDARs at CF-PC synapses during the spontaneous complex spike (CS) activity in cerebellar cortex in urethane-anesthetized mice, by *in vivo* whole-cell recording technique and pharmacological methods. Under current-clamp conditions, cerebellar surface application of NMDA (50 μM) induced an increase in the CS-evoked pause of simple spike (SS) firing accompanied with a decrease in the SS firing rate. Under voltage-clamp conditions, application of NMDA enhanced the waveform of CS-evoked inward currents, which expressed increases in the area under curve (AUC) and spikelet number of spontaneous CS. NMDA increased the AUC of spontaneous CS in a concentration-dependent manner. The EC_50_ of NMDA for increasing AUC of spontaneous CS was 33.4 μM. Moreover, NMDA significantly increased the amplitude, half-width and decay time of CS-evoked after-hyperpolarization (AHP) currents. Blockade of NMDARs with D-(-)-2-amino-5-phosphonopentanoic acid (D-APV, 250 μM) decreased the AUC, spikelet number, and amplitude of AHP currents. In addition, the NMDA-induced enhancement of CS activity could not be observed after α-amino-3-hydroxy-5-methyl-4-isoxazolepropionic acid receptors were blocked. The results indicated that NMDARs of CF-PC synapses contributed to the spontaneous CS activity by enhancing CS-evoked inward currents and AHP currents.

## Introduction

*N*-Methyl-D-aspartate receptors (NMDARs) are widely expressed in the central nervous system, which play important roles in synaptic transmission and synaptic plasticity in adult cerebellar cortex (Dingledine et al., 1999; Cull-Candy et al., 2001). There are three main subunits of NMDA receptor have been identified, NMDAR_1_, NMDAR_2_, and NMDAR_3_ (Cull-Candy et al., 2001; Neyton and Paoletti, 2006). Earlier studies have illustrated that cerebellar Purkinje cells (PCs) in immature rodents express NMDAR_1_ and NMDAR_2_ (Dupont et al., 1987; Rosenmund et al., 1992; Cull-Candy et al., 1998), and electrophysiological recordings have concluded that functional NMDA receptors no longer express after the first postnatal week (Konnerth et al., 1990; Llano et al., 1991; Farrant et al., 1994). however, NMDAR_1_ and NMDAR_2_ subunits have been later found in PCs of adult mice (Nakagawa et al., 1996; Hafidi and Hillman, 1997; Thomson, 2000), and NMDA evokes responses have been recorded in cerebellar PCs of the adult rat and mouse under *in vitro* conditions (Dupont et al., 1987; Billard and Pumain, 1989). Moreover, we recently found that cerebellar surface application of NMDA failed to excite PCs but increased spike firing of molecular layer interneurons (MLIs), resulting in an inhibition in spontaneous SS firing rate of PCs *in vivo* in mice (Liu et al., 2014).

Cerebellar cortical PCs receive information via the mossy fiber-parallel fiber (MF-PF) and the climbing fiber (CF) pathways. Activation of the CF evokes a powerful and infrequent all-or none complex spikes (CSs), consisting of a brief burst of ordinary action potentials and Ca^2+^-dependent dendritic spikes (Eccles et al., 1966), which plays a central role in behaviors by generating excitatory post-synaptic potentials (EPSPs) to PCs (Simpson et al., 1996). CS is thought to represent a critical signal for the operation of the cerebellar cortex, conveying both timing information (Welsh and Llinás, 1996) and triggering synaptic plasticity (Gilbert and Thach, 1977; Hansel et al., 2001; Ito, 2001). Under *in vivo* conditions, CF discharge can control the frequency and pattern of PC spike output by punctuating tonic activity with a variable duration pauses, slow the frequency of simple spike (SS) discharge (Ebner and Bloedel, 1981; Ebner et al., 1983; Barmack and Yakhnitsa, 2003; Cerminara and Rawson, 2004).

*N*-methyl-D-aspartate receptors have been suggested that contributed less to CF activation-mediate inward currents (Otis et al., 1997; Auger and Attwell, 2000). However, NMDARs were post-synaptically expressed at CF-PC synapses and contributed to the waveform of the CF stimulation-induced CS in cerebellar slices of adult mice (Piochon et al., 2007). The NMDARs-mediated CF-PC synaptic transmission develops with maturation of PCs, which is hardly detectable before postnatal day 21, and reach full expression levels at 8 weeks after birth (Piochon et al., 2007; Renzi et al., 2007). Using NMDAR2D*-*/*-*mice, it has been found NMDARs-mediated excitatory post-synaptic currents (EPSCs) could be evoked by CF stimulation via activation of NMDAR2A subunit (Renzi et al., 2007). In addition, the functional NMDARs in PCs of 2–3-months-old rats have also been demonstrated in contribution of CF-evoked EPSCs (Bidoret et al., 2009).

Collectively, NMDARs are post-synaptically expressed at CF-PC synapses and contributed to the waveform of the CF stimulation-induced CS under *in vitro* conditions. However, the role of CF-PC NMDARs in intact cerebellar cortex of living mouse is currently unclear.

## Materials and Methods

### Anesthesia and Surgical Procedures

The anesthesia and surgical procedures have been described previously (Chu et al., 2011a,b). In brief, the experimental procedures were approved by the Animal Care and Use Committee of Jilin University and were in accordance with the animal welfare guidelines of the U.S. National Institutes of Health. The permit number is SYXK (Ji) 2007-0011. Thirty nine adult (6–8-weeks-old) HA/ICR mice were anesthetized with urethane (1.3 g/kg body weight i.p.), and each mouse only recorded one PC in this study (six mice failed to obtain whole-cell recordings of PCs). A watertight chamber was created and a 1–1.5 mm craniotomy was drilled to expose the cerebellar surface corresponding to Vermis VI–VII. The brain surface was constantly superfused with oxygenated artificial cerebrospinal fluid (ACSF: 125 mM NaCl, 3 mM KCl, 1 mM MgSO_4_, 2 mM CaCl_2_, 1 mM NaH_2_PO_4_, 25 mM NaHCO_3_, and 10 mM D-glucose) with a peristaltic pump (Gilson Minipulse 3; Villiers, Le Bel, France) at 0.4 ml/min. Rectal temperature was monitored and maintained at 37.0 ± 0.2°C using body temperature equipment.

### Electrophysiological Recording and Drug Application

*In vivo* cell-attached and whole-cell patch-clamp recordings from PCs were performed with an Axopatch-200B amplifier (Molecular Devices, Foster City, CA, USA). The signals of PC whole-cell recordings were acquired through a Digidata 1440 series analog-to-digital interface on a personal computer using Clampex 10.3 software. Patch pipettes were made with a puller (PB-10; Narishige, Tokyo, Japan) from thick-wall borosilicate glass (GD-1.5; Narishige). Patch electrodes (4–6 MΩ) contained a solution of the following composition (in mM): potassium gluconate 120, HEPES 10, EGTA 1, KCl 5, MgCl_2_ 3.5, NaCl 4, biocytin 8, Na_2_ATP 4, and Na_2_GTP 0.2 (pH 7.3 with KOH, osmolarity adjusted to 300 mOsm). The whole-cell recordings from PCs were performed at depths 150–200 μm under pia mater membrane, and identified by regular spontaneous SS accompanied with irregular CS, and confirmed by biocytinhistochemistry (Chu et al., 2011a). The series resistances were in a range of 10–40 MΩ, compensated by 80%. Membrane voltage and current were filtered at 2 kHz, digitized at 20 kHz. The reagents included *N*-methyl-D-aspartate (NMDA), D-(-)-2-amino-5-phosphonopentanoic acid (D-APV), SR95531, hydrobromide (6-imino-3-(4-methoxyphenyl)-1(6H)-pyridazinebutanoic acid hydrobromide) and NBQX (2,3-dioxo-6-nitro-1,2,3,4-tetrahydrobenzo[f] quinoxaline-7- sulfonamide) were purchased from Sigma-Aldrich (Shanghai, China). The drugs were dissolved in ACSF, and applied directly onto the cerebellar surface for 5 min by a peristaltic pump (0.5 ml/min).

### Histochemistry

After the experiments, the whole brain was removed and fixed in 4% paraformaldehyde in 0.1 PBS (pH 7.4) at 4°C for 24 h. Slices were cut in the sagittal plane at 200 μm using a vibratome (NVSLM1, Campden Instruments LTD, Loughborough, England), and washed with PBS. The tissue was reacted overnight with an avidin-biotin complex (ABC Elite kit; Vector Laboratories, Burlingame, CA, USA) at 4°C. Finally, biocytin binding was visualized by 3,3′-diaminobenzidine tetrahydrochloride histochemistry.

### Data Analysis

The electrophysiological data were analyzed using Clampfit 10.3 software. Spontaneous activities of SS and CS were calculated from a train of interspike intervals recorded for 100 s of baseline, in the presence of drugs and washout (20 min). All the data were normalized with baseline and used for analyses. Values are expressed as the mean ± SEM. Student’s paired *t*-test and one-way ANOVA (SPSS software; Chicago, IL, USA) were used to determine the level of statistical significance between groups of data. *P*-values below 0.05 were considered to indicate a statistically significant difference between experimental groups.

## Results

### Effects of NMDA on CS Activity of the Cerebellar PCs

**FIGURE 1:**
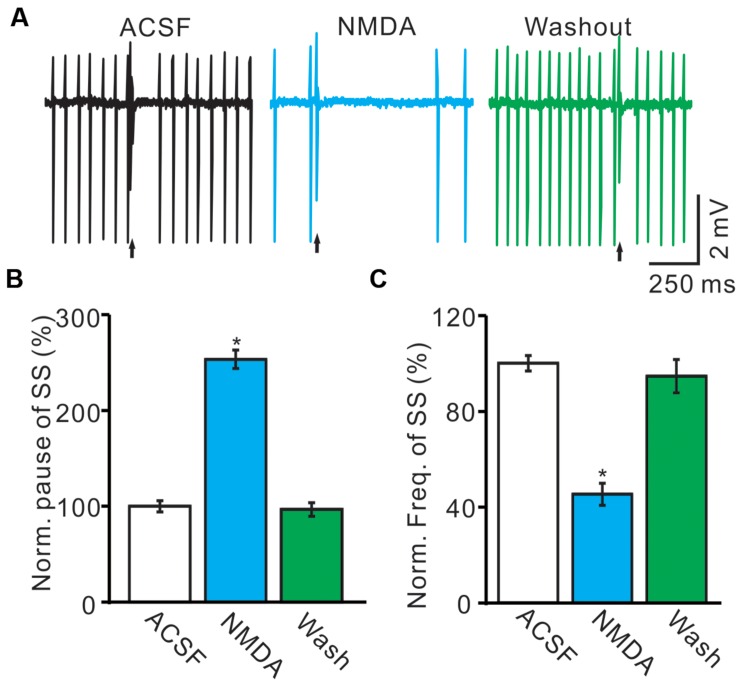
**Effects of NMDA on the spontaneous SS and CS of cerebellar PCs.** **(A)** Representative traces showing that the spontaneous activity from of a PC in treatments: ACSF, NMDA (50 μM) with overlapped trace of ACSF, and washout. Arrows indicated CS. **(B)** Summary of data showing the normalized SS firing rate of each treatment. **(C)** Bar graph showing the normalized pause of SS in ACSF, NMDA and washout. ^∗^*P* < 0.05; *n* = 8.

Under cell-attached recording conditions, PCs were identified by regular SS firing and the presence of irregular CS (Ito, 1984; Chu et al., 2011b). These PCs exhibited irregular CS firing at mean rates of 0.9 ± 0.3 Hz (*n* = 8) recorded in cerebellar cortex Vermis VII. The highest and lowest rates of CS firing were 1.9 and 0.1 Hz, respectively. Consistent with our previous study (Liu et al., 2014), cerebellar surface perfusion of 50 μM NMDA for 5 min induced depression in the frequency of SS firing rate of PCs (**Figure 1A**), the normalized SS firing rate was 44.2 ± 4.8% of baseline (100.0 ± 3.2%; *n* = 8; *P* < 0.001; **Figure 1B**). However, NMDA induced a significant increase in pause of SS firing (**Figures 1A,C**), the normalized pause of SS firing was 253.4 ± 9.5% of baseline (100.0 ± 3.2%; *n* = 8; *P* < 0.001; **Figure 1C**). The effect of NMDA on SS firing rate of PCs was recovered after washout for 20 min. These results suggested that extracellular application of NMDA modulated CS activity of the PCs.

**FIGURE 2:**
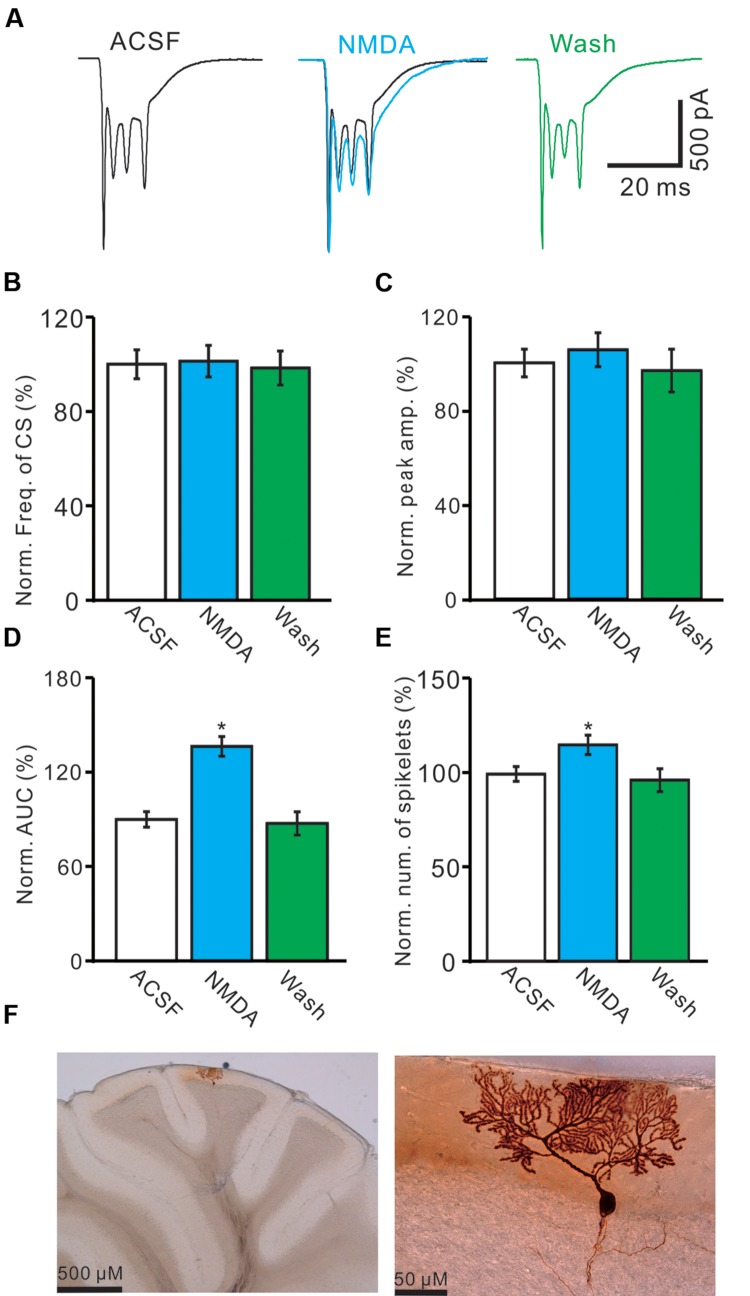
**Effects of NMDA on waveform properties of inward currents evoked by spontaneous CS.** **(A)** Representative traces showing the properties of CS-evoked inward currents of PC in ACSF, NMDA (50 μM; blue) with overlapped trace of ACSF (black), and washout. **(B)** Pooled data showing the normalized frequency of spontaneous CS. **(C)** Summary of data shows the effects of NMDA on the normalized peak amplitude of CS. **(D,E)** Bar graphs show the normalized AUC **(D)** and spikelet number **(E)** of CS in ACSF, NMDA and washout. **(F)** Microphotograph showing the recorded PC filled with biocytin. ^∗^*P* < 0.05; *n* = 8.

**FIGURE 3:**
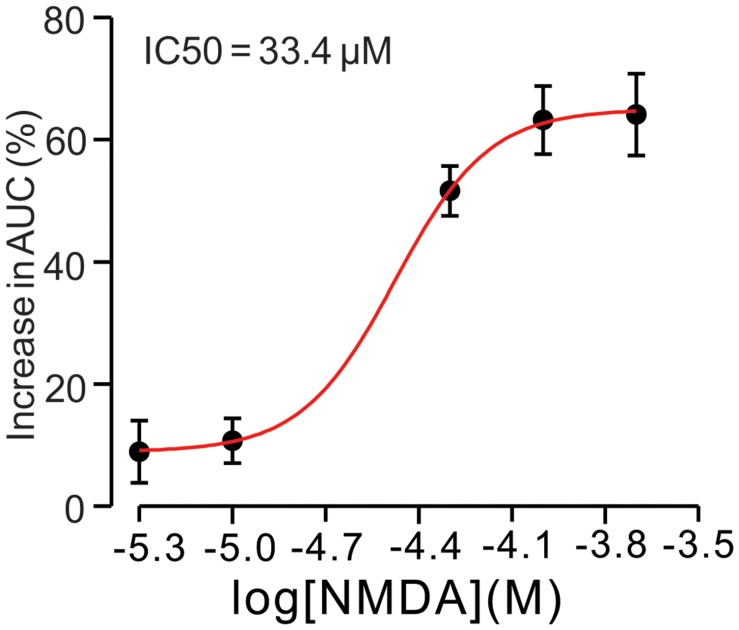
***N*-methyl-D-aspartate receptor (NMDA)-induced increase in AUC of CS is in a dose-dependent manner.** Bath application of NMDA enhanced the AUC of CS in a dose-dependent manner (IC_50_ = 33.4 μM).The number of the recorded PCs tested for each concentration is indicated near the bars.

For understand the effect of NMDA on CS-evoked membrane currents of PCs, we further performed *in vivo* whole-cell patch-clamp recording in a total 25 cerebellar PCs. Under voltage-clamp recording conditions (V_hold_ = -70 mV), the spontaneous CS expressed strong inward currents with high frequency spiklets, followed by outward currents (**Figures 2A,F**). The mean value of outward currents was 107.4 ± 4.7 pA (*n* = 25; not shown). The number of spikelets was 2–5 and the mean frequency of them was 406 ± 6.5 Hz (*n* = 25; not shown). Cerebellar surface perfusion of NMDA (50 μM) did not significantly change the frequency (**Figures 2A,B**) and peak amplitude (**Figures 2A,C**) of spontaneous CS, but increased the area under curve (AUC) of CS-evoked inward currents by 151.6 ± 6.9% of baseline (ACSF: 100.0 ± 5.5%; *n* = 8; *P* < 0.05; **Figures 2A,D**). NMDA also significantly increased number of spikelets to 115.6 ± 5.2% of baseline (ACSF: 100.0 ± 3.9%; *n* = 8; *P* < 0.05; **Figures 2A,E**). NMDA increased the AUC of CS in a concentration-dependent manner (**Figure 3**). AUC of CS was significantly increased by 8.9 ± 5.1% of control (ACSF) with 5 μM NMDA (*n* = 6; **Figure 3**), and the 50% effective concentration (EC_50_) was 33.4 μM. AUC of CS was significantly (*P* < 0.001) increased by 64.1 ± 6.7% of baseline with 200 μM NMDA (*n* = 5; **Figure 3**). Consistent with previous studies (Piochon et al., 2007, 2010; Renzi et al., 2007), our results indicated that extracellular application of NMDA dose-dependently enhanced the strength of CS activity *in vivo* in mice.

### NMDA Enhanced CS-Evoked After-Hyperpolarzation (AHP) Currents

**FIGURE 4:**
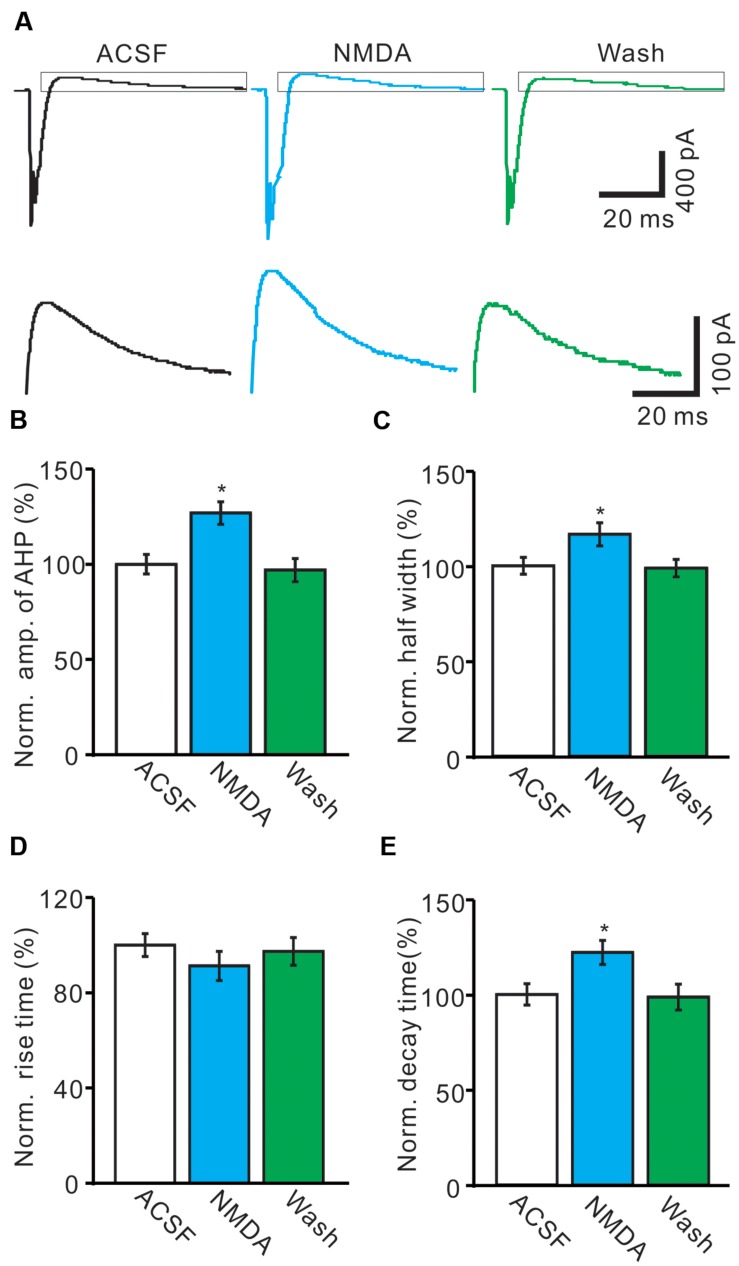
***N*-methyl-D-aspartate receptor modulates the waveform of CS-evoked AHP currents.** **(A)** Upper, Representative traces showing the spontaneous CS-evoked current waveforms of a PC in ACSF, NMDA (50 μM) and washout. Lower shows the enlarged traces of AHP currents of upper panel (quadrangle). **(B,D)** Bar graphs showing the normalized amplitude **(B)**, half-width **(C)**, rise time **(D)**, and decay time **(E)** of AHP in ACSF, NMDA and washout. ^∗^*P* < 0.05; *n* = 8.

The CS evoked outward currents have been identified as AHP currents, which have been assumed to be critical for regulation of the frequency and pattern of PC SS output (McKay et al., 2007), we therefore examined that effect of NMDA on properties of AHP currents. As shown in **Figure 4**, application of NMDA significantly affected the waveforms of the spontaneous CS firing. In the presence of NMDA (50 μM), the normalized amplitude of AHP currents was increased to 126.9 ± 5.9% of baseline (ACSF: 100.0 ± 5.1%; *n* = 8; *P* < 0.05; **Figures 4A,B**). The normalized half-width of AHP currents was increased to 116.4 ± 6.1% of baseline (ACSF: 100.0 ± 4.4%; *n* = 8; *P* < 0.05; **Figures 4A,C**). In addition, NMDA significantly decreased the rise time of AHP currents to 86.3 ± 6.1% of baseline (ACSF: 100.0 ± 4.8%; *n* = 8; *P* < 0.05; **Figures 4A,D**). However, the normalized decay time of AHP currents was significantly increased to 122.1 ± 6.3% of baseline (ACSF: 100.0 ± 5.6%; *n* = 8; *P* < 0.05; **Figures 4A,E**). These results indicated that NMDA enhanced the amplitude and the dynamic properties of AHP currents *in vivo* in mice.

### Blockade of NMDA Receptor Attenuated the Activity of Spontaneous CS

**FIGURE 5:**
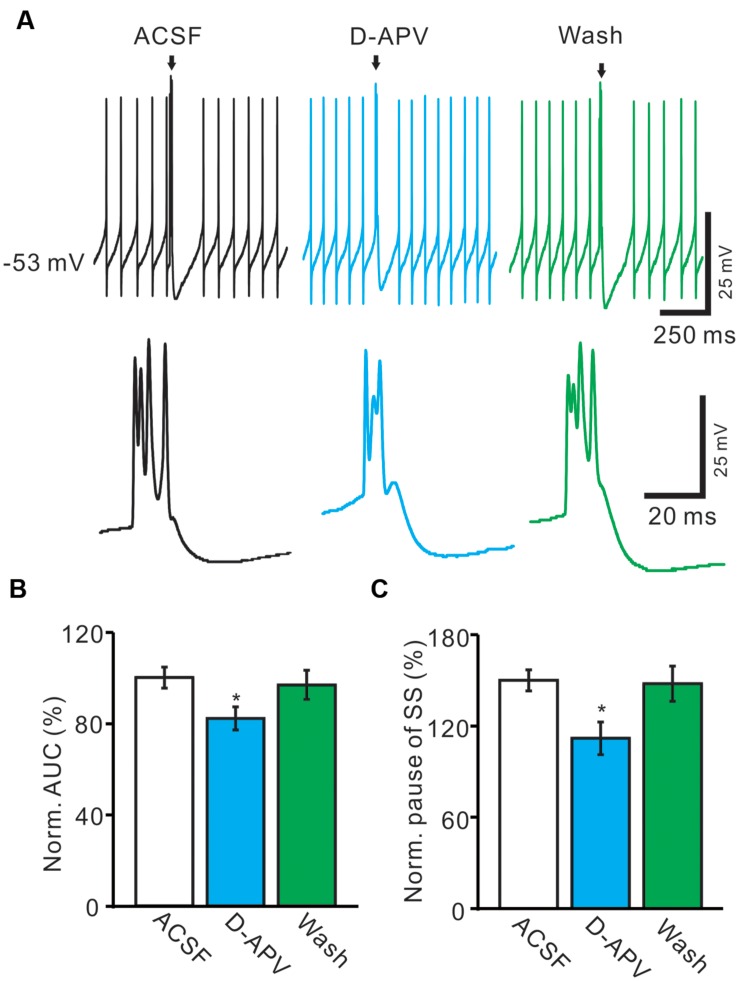
**Blockade of NMDA receptor attenuated the waveform of spontaneous CS.** **(A)** Upper, representative traces showing the spontaneous SS and CS activities of a PC in ACSF, D-APV (250 μM) and washout. Lower, the enlarged traces of CS of upper panel (arrows). **(B)** Bar graphs show the normalized pause of SS in ACSF, D-APV and washout. **(C)** Summary of data shows the effects of D-APV on the normalized number of CS spikelets. **(D)** Pooled data showing the normalized AUC of CS in ACSF, D-APV and washout. **(E)** Summary of data showing the normalized amplitude of AHP potentials in ACSF, NMDA and washout. ^∗^*P* < 0.05; *n* = 6.

We further used an NMDA antagonist, D-APV (250 μM) to determine whether the functional NMDARs were activated during the spontaneous CS spike firing in living mouse. In the presence of D-APV, the normalized pause of SS firing was decreased to 82.1 ± 5.1% of baseline (100.0 ± 4.7%; *n* = 6; *P* < 0.001; **Figure 5B**), and the normalized number of spikelets was decreased to 86.4 ± 6.3% of baseline (ACSF: 100.0 ± 4.6%; *n* = 6; *P* < 0.05; **Figures 5A,C**). Moreover, D-APV induced a significant decrease in the AUC of CS-evoked excitatory potentials to 74.6 ± 7.1% of baseline (ACSF: 100.0 ± 4.6%; *n* = 6; *P* < 0.05; **Figures 5A,D**), and the normalized amplitude of AHP potential was decreased to 85.1 ± 6.7% of baseline (ACSF: 100.0 ± 5.8%; *n* = 6; *P* < 0.05; **Figures 5A,E**). These results indicated that blockade of CF-PC synaptic NMDA receptor attenuated the strength of spontaneous CS activity in living mice.

### Blockade AMPA Receptors Abolished Spontaneous CS Firing and NMDARs Activity

**FIGURE 6:**
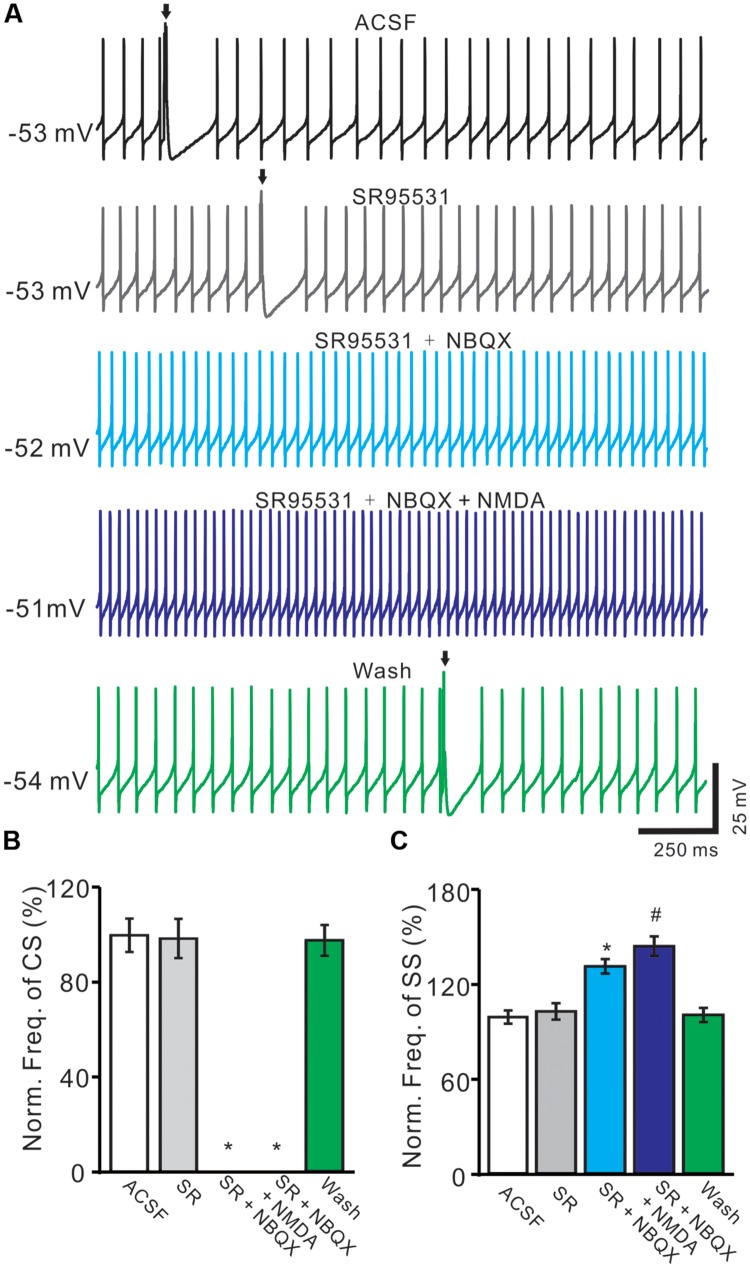
**Blockade AMPARs abolished spontaneous CS firing and NMDARs activity.** **(A)** Representative traces showing the spontaneous SS and CS (arrows) activities of a PC in ACSF, SR95531 (20 μM), SR95531 (20 μM) + NBQX (50 μM), SR95531 + NBQX + NMDA (50 μM) and washout. **(B,C)** Bar graph shows the normalized frequency of CS **(B)** and frequency of SS **(C)** in ACSF, SR95531, SR95531+ NBQX, SR95531 + NBQX + NMDA and washout. ^∗^*P* < 0.05 vs. ACSF; ^#^*P* < 0.05 vs. SR + NBQX. *n* = 6.

For understanding the functional relationships between NMDARs and α-amino-3-hydroxy-5-methyl-4-isoxazolepropionic acid receptors (AMPARs) during the spontaneous CS activity, we employed AMPARs antagonist, NBQX. In these experiments, we also used GABA_A_ receptor antagonist, gabazine (SR95531, 20 μM) to prevent inhibitory effects of MLIs. In the presence of gabazine, NBQX (50 μM) completely blocked the spontaneous CS activity (**Figure 6A**). The normalized frequency of CS (0 ± 0%) was significant lower that in control conditions (ACSF: 100.0 ± 7.1%; *n* = 6; *P* < 0.05 vs. control; **Figures 6A,B**). Additional application of NMDA could not restored the CS activity (**Figures 6A,B**). These results indicated that the spontaneous activity of CS was mediated predominantly by AMPARs, but activation of NMDARs might enhance the spontaneous CS firing activity of PCs.

The frequency of SS firing was unaffected by blocking of GABA_A_ receptors activity, but blockade CS activity with NBQX induced an increase in SS firing rate to 132.4 ± 4.6% of control (100 ± 4.2%; *n* = 6; *P* < 0.05 vs. control; **Figures 6A,C**). Additional application of NMDA induced a further increase in SS firing rate to 145.3 ± 6.2% of control (100 ± 4.2%; *n* = 6; *P* < 0.05 vs. control; **Figures 6A,C**). These results indicated that blockade CS activity increased SS firing rate of PCs in cerebellar folium Vermis I-II, suggesting that CS activity modulated SS spike activity *in vivo* in mice (Ebner and Bloedel, 1981; Ebner et al., 1983; Barmack and Yakhnitsa, 2003; Cerminara and Rawson, 2004).

## Discussion

In the present study, we mainly found that extracellular application of NMDA dose-dependently enhanced the strength of spontaneous CS activity, whereas NMDA blocker attenuated the strength of CS activity *in vivo* in mice. Our results indicated that NMDA receptors of CF-PC synapses contributed to spontaneous CS activity, suggesting that post-synaptic NMDA receptors of CF-PC synapses play important roles in the regulation of spontaneous CS activity *in vivo* in mice.

### Properties of the Spontaneous CS Discharge *In vivo*

Under *in vivo* conditions, CF discharge can control the frequency and pattern of PC spike output by punctuating tonic activity with a variable duration pauses, slow the frequency of SS discharge (Ebner and Bloedel, 1981; Ebner et al., 1983; Barmack and Yakhnitsa, 2003; Cerminara and Rawson, 2004; Loewenstein et al., 2005). Removal of CF inputs uncovers an increase in SS discharge rate or even a slow oscillatory pattern of background and PC SS discharge that returns to tonic activity when CF activity is reinstated (Colin et al., 1980; Montarolo et al., 1982; Simpson et al., 1996; Cerminara and Rawson, 2004). The ability for CF inputs to restore tonic discharge *in vivo* is considered partly due to a block of the mechanisms that give rise to an intrinsic trimodal pattern of PC output (McKay et al., 2007). Therefore, CS is thought to represent a critical signal for the operation of the cerebellar cortex, conveying both timing information (Welsh and Llinás, 1996) and triggering synaptic plasticity (Gilbert and Thach, 1977; Hansel et al., 2001; Ito, 2001).

The possible mechanisms underlying CFs activity are usually investigated by simulation of CF to evoke a CS-like depolarization under *in vivo* and *in vitro* conditions. However, the stimulation-evoked CS-like depolarization could not fully replicate the actions of a CS evoked by synaptic input under *in vivo* conditions (Reynolds and Hartell, 2000). Electrical stimulation of CF evoked CS followed by a slow after-depolarization (ADP) and an AHP (Schmolesky et al., 2005), whereas the spontaneous CF activity evoked CS followed by AHP *in vivo* in mice. Under *in vitro* conditions, strong electrical stimulation of CFs afferents induced ADP which has been considered to be mediated by NMDARs (Piochon et al., 2007) or by metabotropic glutamate receptors (Yuan et al., 2007). However, spontaneous activity of CS *in vivo* could induce a brief depolarization of PCs, resulting in rapid Ca^2+^–Na^+^ bursts and prolonged increases in Ca^2+^ concentrations (Miyakawa et al., 1992; Maeda et al., 1999). The Ca^2+^ flowing into PCs triggers a net outward current through Ca^2+^-dependent K^+^ currents, which exhibited AHP after CS firing. The CS-evoked AHP has been assumed to be a key effecter of the CF-evoked pause and the spike firing activity of SS (Hounsgaard and Midtgaard, 1989).

### NMDARs Contribute to Spontaneous CS Discharge in Cerebellar PCs

*N*-methyl-D-aspartate receptors are widely expressed in the central nervous system, playing critical roles in synaptic transmission, synaptic plasticity and synaptogenesis (Dingledine et al., 1999; Cull-Candy et al., 2001; Van Zundert et al., 2004). Under *in vitro* conditions, it has been assumed that NMDARs contribute less to CF stimulation evoked inward currents (Otis et al., 1997; Auger and Attwell, 2000). Recently, however, it was demonstrated that NMDARs contributed to the CF response *in vitro* in mice (Piochon et al., 2007, 2010). In this study, we showed that extracellular application of NMDA enhanced the waveform of spontaneous CS-evoked inward currents, which expressed increases in the AUC and spikelets of CS, suggesting that NMDA enhanced CF-PC synaptic transmission via activation of NMDARs at CF-PC synapses. Our results are supported by several previous studies (Piochon et al., 2007, 2010; Renzi et al., 2007; Bidoret et al., 2009). First, NMDARs contain NMDAR2A and/or NMDAR2-B subunits are indeed post-synaptically expressed at CF-PC synapses in adult mice, which contributed to the waveform of CS induced by the CF stimulation in cerebellar slices (Piochon et al., 2007). Dendritic patch-clamp recording showed that CR responses were reduced by NMDARs blocker, D-APV (Piochon et al., 2010). Furthermore, CF stimulation can evoke NMDARs-mediated EPSCs at CF-PC synapses, which is mediated mainly by NMDAR_2A_-containing receptors in adult NMDAR_2D_ knockout mice (Renzi et al., 2007). Moreover, functional NMDARs in PCs of 2–3-months-old rats have also been demonstrated in CF-evoked EPSCs (Bidoret et al., 2009). In addition, our data showed that application of NMDARs antagonist attenuated the waveform of spontaneous CS, which expressed decreases in the AUC and spkelet number of the spontaneous CS, confirming that CF-PC synaptic NMDARs contributed to the spontaneous CS spike firing in living mice.

In addition, our present results showed that activating and blocking NMDARs modulated the pause of SS firing, suggesting that PF-PC synaptic NMDARs contributed to SS output of PCs via a post-synaptic mechanism. The CS-evoked AHP has been assumed to be a key effecter of the CF-evoked pause and the spike firing activity of SS (Hounsgaard and Midtgaard, 1989). Our present results showed that NMDA significantly increasing the amplitude, half-width and decay time of the spontaneous CS-evoked AHP currents, whereas D-APV attenuated the amplitude of CS-evoked AHP currents. These results suggested that CF-PC synaptic NMDARs contributed to SS output of PCs via modulation of AHP activity under *in vivo* conditions. It has been reported that CF stimulation evoked inward currents which are mediated predominantly by AMPARs (Otis et al., 1997), and the AMPA receptor antagonist, NBQX abolished the CS and the its associated calcium transient in mouse cerebellar slices (Yuan et al., 2007). Consistent with previous studies (Otis et al., 1997; Yuan et al., 2007), our results showed that the spontaneous CS firing was abolished by AMPARs antagonist, indicated that the spontaneous activity of CSs was mediated predominantly by AMPARs under physiological conditions. However, NMDA could not reverse the CS activity in the presence of AMPARs antagonist, suggesting that the spontaneous CSs firing-evoked activity of NMDARs was dependent on the AMPARs activation. It was demonstrates that the CS-induced depolarization is sufficient to relieve the Mg^2+^ block of NMDA-Rs and to allow them to be activated during the CS firing (Piochon et al., 2007). In addition, stimulation CF evoked NMDA-mediated CF-EPSP in absence of Mg^2+^ and AMPARs activity, which was completely abolished by D-APV and Mg^2+^ in mouse cerebellar PCs *in vitro* (Piochon et al., 2007). These results indicate that activation of CF could evoke NMDA receptor-mediated currents in absence of AMPARs activation, but NMDA receptors blocker, Mg^2+^ should be removed. Collectively, our present results suggest that the spontaneous activity of CF afferents induces glutamate release into CF-PC synapses, which activates post-synaptic AMPARs and resulting in a strong depolarization of post-synaptic membrane (inward currents) *in vivo* in mice. This strong depolarization removes NMDARs blocker, Mg^2+^, therefore, Ca^2+^ influx into PC somas from NMDARs and induces an increase in intracellular Ca^2+^ level (Miyakawa et al., 1992; Maeda et al., 1999). The increase of Ca^2+^ concentration could further activate Ca^2+^-dependent K^+^ currents, resulting in an enhance of AHP activity and a increase in pause of SS firing (Hounsgaard and Midtgaard, 1989).

### Physiological Significant of NMDARs Activity in CF-PC Synapses

The predominantly studied form of cerebellar plasticity is long-term depression (LTD) at PF to PC synapses, which results from PF and CF co-activation and is assumed to mediate forms of cerebellar motor learning (Ito et al., 1982; Ito, 2002). CF activity provides widespread Ca^2+^ transients (Ross and Werman, 1987; Konnerth et al., 1992; Miyakawa et al., 1992), which are required for PF-LTD induction (Sakurai, 1990; Konnerth et al., 1992). In principle, CF-PC synaptic NMDARs could mediate a phasic influx of Ca^2+^ with a spatiotemporal profile potentially different from that of Ca^2+^ elevation associated with CF-evoked CSs (Hartmann and Konnerth, 2005). Importantly, probability of LTD and long-term potentiation (LTP) at PF inputs is under control of the CF (Coesmans et al., 2004), which itself undergoes Ca^2+^-dependent LTD (Hansel and Linden, 2000). NMDA receptors are post-synaptically expressed at CF-PC synapses in young adult/adult mice (Piochon et al., 2007), and are required for LTD induction after onset of expression (Piochon et al., 2010). Single pulse stimulation of CF leads to a large all-or-none response in the dendrite (Davie et al., 2008; Ohtsuki et al., 2009), which is strong enough to activate NMDARs signaling, and ultimately evokes a CS in PC somatic recordings *in vitro*, and these effects are expected to favor Ca^2+^ entry in the dendrites as well as their propagation (Schmolesky et al., 2002). In addition, NMDA signaling may provide the calcium transients that activate calcium-dependent SK2-type potassium conductance that underlie the pause. Plasticity of these SK2 channels mediates plasticity of the pause itself (Grasselli et al., 2016).

## Author Contributions

Conception and design of experiments: C-PC, D-LQ. Performance of experiments: HL,YL. Analysis of data: C-PC, D-LQ. Contribution of reagents/materials/analysis tools: Y-HB. Writing of the manuscript: C-PC, D-LQ.

## Conflict of Interest Statement

The authors declare that the research was conducted in the absence of any commercial or financial relationships that could be construed as a potential conflict of interest.

## References

[B1] Auger C. Attwell D. (2000). Fast removal of synaptic glutamate by postsynaptic transporters. *Neuron* 28 547–558. 10.1016/S0896-6273(00)00132-X 11144363

[B2] Barmack N. H. Yakhnitsa V. (2003). Cerebellar climbing fibers modulate simple spikes in Purkinje cells. *J. Neurosci.* 23 7904–7916. 12944521 10.1523/JNEUROSCI.23-21-07904.2003 PMC6740591

[B3] Bidoret C. Ayon A. Barbour B. Casado M. (2009). Presynaptic NR2A-containing NMDA receptors implement a high-pass filter synaptic plasticity rule. *Proc. Natl. Acad. Sci. U.S.A.* 106 14126–14131. 10.1073/pnas.0904284106 19666514 PMC2729031

[B4] Billard J. M. Pumain R. (1989). Loss of N-methyl-D-aspartate sensitivity of cerebellar Purkinje cells after climbing fiber deafferentation. *Neurosci. Lett.* 106 199–204. 10.1016/0304-3940(89)90226-7 2555747

[B5] Cerminara N. L. Rawson J. A. (2004). Evidence that climbing fibers control an intrinsic spike generator in cerebellar Purkinje cells. *J. Neurosci.* 24 4510–4517. 10.1523/JNEUROSCI.4530-03.2004 15140921 PMC6729399

[B6] Chu C. P. Bing Y. H. Liu Q. R. Qiu D. L. (2011a). Synaptic responses evoked by tactile stimuli in Purkinje cells in mouse cerebellar cortex crus II. *PLoS ONE* 6:e22752 10.1371/journal.pone.0022752 PMC3144243 21818384

[B7] Chu C. P. Bing Y. H. Qiu D. L. (2011b). Sensory stimulus evokes inhibition rather than excitation in cerebellar Purkinje cells in vivo in mice. *Neurosci. Lett.* 487 182–186. 10.1016/j.neulet.2010.10.018 20965231

[B8] Coesmans M. Weber J. T. De Zeeuw C. I. Hansel C. (2004). Bidirectional parallel fiber plasticity in the cerebellum under climbing fiber control. *Neuron* 44 691–700. 10.1016/j.neuron.2004.10.031 15541316

[B9] Colin F. Manil J. Desclin J. C. (1980). The olivocerebellar system. I. Delayed and slow inhibitory effects: an overlooked salient feature of cerebellar climbing fibers. *Brain Res.* 187 3–27. 10.1016/0006-8993(80)90491-6 7357475

[B10] Cull-Candy S. Brickley S. Farrant M. (2001). NMDA receptor subunits: diversity, development and disease. *Curr. Opin. Neurobiol.* 11 327–335. 10.1016/S0959-4388(00)00215-4 11399431

[B11] Cull-Candy S. G. Brickley S. G. Misra C. Feldmeyer D. Momiyama A. Farrant M. (1998). NMDA receptor diversity in the cerebellum: identification of subunits contributing to functional receptors. *Neuropharmacology* 37 1369–1380. 10.1016/S0028-3908(98)00119-1 9849672

[B12] Davie J. T. Clark B. A. Häusser M. (2008). The origin of the complex spike in cerebellar Purkinje cells. *J. Neurosci.* 28 7599–7609. 10.1523/JNEUROSCI.0559-08.2008 18650337 PMC2730632

[B13] Dingledine R. Borges K. Bowie D. Traynelis S. F. (1999). The glutamate receptor ion channels. *Pharmacol. Rev.* 51 7–62. 10049997

[B14] Dupont J. L. Gardette R. Crepel F. (1987). Postnatal development of the chemosensitivity of rat cerebellar Purkinje cells to excitatory amino acids. *Dev. Brain Res.* 34 59–68. 10.1016/0165-3806(87)90195-7 2887259

[B15] Ebner T. J. Bloedel J. R. (1981). Role of climbing fiber afferent input in determining responsiveness of Purkinje cells to mossy fiber inputs. *J. Neurophysiol.* 45 962–971. 7241180 10.1152/jn.1981.45.5.962

[B16] Ebner T. J. Yu Q. X. Bloedel J. R. (1983). Increase in Purkinje cell gain associated with naturally activated climbing fiber input. *J. Neurophysiol.* 50 205–219. 6308180 10.1152/jn.1983.50.1.205

[B17] Eccles J. C. Llinas R. Sasaki K. (1966). The excitatory synaptic action of climbing fibres on the Purkinje cells of the cerebellum. *J. Physiol.* 182 268–296. 10.1113/jphysiol.1966.sp007824 5944665 PMC1357472

[B18] Farrant M. Feldmeyer D. Takahashi T. Cull-Candy S. G. (1994). NMDA-receptor channel diversity in the developing cerebellum. *Nature* 368 335–339. 10.1038/368335a0 7907398

[B19] Gilbert P. F. C. Thach W. T. (1977). Purkinje cell activity during motor learning. *Brain Res.* 128 309–328. 10.1016/0006-8993(77)90997-0 194656

[B20] Grasselli G. He Q. Wan V. Adelman J. P. Ohtsuki G. Hansel C. (2016). Activity-dependent plasticity of spike pauses in cerebellar Purkinje cells. *Cell Rep.* 14 2546–2553. 10.1016/j.celrep.2016.02.054 26972012 PMC4805497

[B21] Hafidi A. Hillman D. E. (1997). Distribution of glutamate receptors GluR 2/3 and NR1 in the developing rat cerebellum. *Neuroscience* 81 427–436. 10.1016/S0306-4522(97)00140-1 9300432

[B22] Hansel C. Linden D. J. (2000). Long-term depression of the cerebellar climbing fiber-Purkinje neuron synapse. *Neuron* 26 473–482. 10.1016/S0896-6273(00)81179-4 10839365

[B23] Hansel C. Linden D. J. D’Angelo E. (2001). Beyond parallel fiber LTD: the diversity of synaptic and non-synaptic plasticity in the cerebellum. *Nat. Neurosci.* 4 467–475. 11319554 10.1038/87419

[B24] Hartmann J. Konnerth A. (2005). Determinants of postsynaptic Ca2+ signaling in Purkinje neurons. *Cell Calcium* 37 459–466. 10.1016/j.ceca.2005.01.014 15820394

[B25] Hounsgaard J. Midtgaard J. (1989). Synaptic control of excitability in turtle cerebellar Purkinje cells. *J. Physiol.* 409 157–170. 10.1113/jphysiol.1989.sp017490 2585289 PMC1190437

[B26] Ito M. (1984). *The Cerebellum and Neural Control.* New York, NY: Raven Press.

[B27] Ito M. (2001). Cerebellar long-term depression: characterization, signal transduction, and functional roles. *Physiol. Rev.* 81 1143–1195. 11427694 10.1152/physrev.2001.81.3.1143

[B28] Ito M. (2002). The molecular organization of cerebellar long-term depression. *Nat. Rev. Neurosci.* 3 896–902. 10.1038/nrn962 12415297

[B29] Ito M. Sakurai M. Tongroach P. (1982). Climbing fibre induced depression of both mossy fibre responsiveness and glutamate sensitivity of cerebellar Purkinje cells. *J. Physiol.* 324 113–134. 10.1113/jphysiol.1982.sp014103 7097592 PMC1250696

[B30] Konnerth A. Dreessen J. Augustine G. J. (1992). Brief dendritic calcium signals initiate long-lasting synaptic depression in cerebellar Purkinje cells. *Proc. Natl. Acad. Sci. U.S.A.* 89 7051–7055. 10.1073/pnas.89.15.7051 1323125 PMC49643

[B31] Konnerth A. Llano I. Armstrong C. M. (1990). Synaptic currents in cerebellar Purkinje cells. *Proc. Natl. Acad. Sci. U.S.A.* 87 2662–2665. 10.1073/pnas.87.7.2662 1969639 PMC53750

[B32] Liu H. Zhao S. N. Zhao G. Y. Sun L. Chu C. P. Qiu D. L. (2014). N-methyl-d-aspartate inhibits cerebellar Purkinje cell activity via the excitation of molecular layer interneurons under in vivo conditions in mice. *Brain Res.* 1560 1–9. 10.1016/j.brainres.2014.03.011 24642274

[B33] Llano I. Leresche N. Marty A. (1991). Calcium entry increases the sensitivity of cerebellar Purkinje cells to applied GABA and decreases inhibitory synaptic currents. *Neuron* 6 565–574. 10.1016/0896-6273(91)90059-9 2015092

[B34] Loewenstein Y. Mahon S. Chadderton P. Kitamura K. Sompolinsky H. Yarom Y. (2005). Bistability of cerebellar Purkinje cells modulated by sensory stimulation. *Nat. Neurosci.* 8 202–211. 10.1038/nn1393 15665875

[B35] Maeda H. Ellis-Davies G. C. R. Ito K. Miyashita Y. Kasai H. (1999). Supralinear Ca2+ signaling by cooperative and mobile Ca2+ buffering in Purkinje neurons. *Neuron* 24 989–1002. 10.1016/S0896-6273(00)81045-4 10624961

[B36] McKay B. E. Engbers J. D. T. Mehaffey W. H. Gordon G. R. J. Molineux M. L. Bains J. S. (2007). Climbing fiber discharge regulates cerebellar functions by controlling the intrinsic characteristics of Purkinje cell output. *J. Neurophysiol.* 97 2590–2604. 10.1152/jn.00627.2006 17267759

[B37] Miyakawa H. Lev-Ram V. Lasser-Ross N. Ross W. N. (1992). Calcium transients evoked by climbing fiber and parallel fiber synaptic inputs in guinea pig cerebellar Purkinje neurons. *J. Neurophysiol.* 68 1178–1189. 1359027 10.1152/jn.1992.68.4.1178

[B38] Montarolo P. G. Palestini M. Strata P. (1982). The inhibitory effect of the olivocerebellar input on the cerebellar Purkinje cells in the rat. *J. Physiol.* 332 187–202. 10.1113/jphysiol.1982.sp014409 7153927 PMC1197394

[B39] Nakagawa S. Watanabe M. Inoue Y. (1996). Altered gene expression of the N-Methyl-d-Aspartate receptor channel subunits in Purkinje cells of the staggerer mutant mouse. *Eur. J. Neurosci.* 8 2644–2651. 10.1111/j.1460-9568.1996.tb01559.x 8996814

[B40] Neyton J. Paoletti P. (2006). Relating NMDA receptor function to receptor subunit composition: limitations of the pharmacological approach. *J. Neurosci.* 26 1331–1333. 10.1523/JNEUROSCI.5242-05.2006 16452656 PMC6675501

[B41] Ohtsuki G. Piochon C. Hansel C. (2009). Climbing fiber signaling and cerebellar gain control. *Front. Cell. Neurosci.* 3:4 10.3389/neuro.03.004.2009 PMC2708967 19597563

[B42] Otis T. S. Kavanaugh M. P. Jahr C. E. (1997). Postsynaptic glutamate transport at the climbing fiber-Purkinje cell synapse. *Science* 277 1515–1518. 10.1126/science.277.5331.1515 9278516

[B43] Piochon C. Irinopoulou T. Brusciano D. Bailly Y. Mariani J. Levenes C. (2007). NMDA receptor contribution to the climbing fiber response in the adult mouse Purkinje cell. *J. Neurosci.* 27 10797–10809. 10.1523/JNEUROSCI.2422-07.2007 17913913 PMC6672834

[B44] Piochon C. Levenes C. Ohtsuki G. Hansel C. (2010). Purkinje cell NMDA receptors assume a key role in synaptic gain control in the mature cerebellum. *J. Neurosci.* 30 15330–15335. 10.1523/JNEUROSCI.4344-10.2010 21068337 PMC2990192

[B45] Renzi M. Farrant M. Cull-Candy S. G. (2007). Climbing-fibre activation of NMDA receptors in Purkinje cells of adult mice. *J. Physiol.* 585 91–101. 10.1113/jphysiol.2007.141531 17901118 PMC2327252

[B46] Reynolds T. Hartell N. A. (2000). An evaluation of the synapse specificity of long-term depression induced in rat cerebellar slices. *J. Physiol.* 527 563–577. 10.1111/j.1469-7793.2000.00563.x 10990541 PMC2270087

[B47] Rosenmund C. Legendre P. Westbrook G. L. (1992). Expression of NMDA channels on cerebellar Purkinje cells acutely dissociated from newborn rats. *J. Neurophysiol.* 68 1901–1905. 1282541 10.1152/jn.1992.68.5.1901

[B48] Ross W. N. Werman R. (1987). Mapping calcium transients in the dendrites of Purkinje cells from the guinea-pig cerebellum in vitro. *J. Physiol.* 389 319–336. 10.1113/jphysiol.1987.sp016659 3681730 PMC1192083

[B49] Sakurai M. (1990). Calcium is an intracellular mediator of the climbing fiber in induction of cerebellar long-term depression. *Proc. Natl. Acad. Sci. U.S.A.* 87 3383–3385. 10.1073/pnas.87.9.3383 2159149 PMC53904

[B50] Schmolesky M. T. De Zeeuw C. I. Hansel C. (2005). Climbing fiber synaptic plasticity and modifications in Purkinje cell excitability. *Prog. Brain Res.* 148 81–94. 10.1016/S0079-6123(04)48008-X 15661183

[B51] Schmolesky M. T. Weber J. T. Zeeuw C. I. Hansel C. (2002). The making of a complex spike: ionic composition and plasticity. *Ann. N. Y. Acad. Sci.* 978 359–390. 10.1111/j.1749-6632.2002.tb07581.x 12582067

[B52] Simpson J. I. Wylie D. R. De Zeeuw C. I. (1996). On climbing fiber signals and their consequence (s). *Behav. Brain Sci.* 19 384–398. 10.1017/S0140525X00081991

[B53] Thomson A. M. (2000). Facilitation, augmentation and potentiation at central synapses. *Trends Neurosci.* 23 305–312. 10.1016/S0166-2236(00)01580-0 10856940

[B54] Van Zundert B. Yoshii A. Constantine-Paton M. (2004). Receptor compartmentalization and trafficking at glutamate synapses: a developmental proposal. *Trends Neurosci.* 27 428–437. 10.1016/j.tins.2004.05.010 15219743

[B55] Welsh J. P. Llinás R. (1996). Some organizing principles for the control of movement based on olivocerebellar physiology. *Prog. Brain Res.* 114 449–461. 10.1016/S0079-6123(08)63380-4 9193160

[B56] Yuan Q. Qiu D. L. Weber J. T. Hansel C. Knöpfel T. (2007). Climbing fiber-triggered metabotropic slow potentials enhance dendritic calcium transients and simple spike firing in cerebellar Purkinje cells. *Mol. Cell. Neurosci.* 35 596–603. 10.1016/j.mcn.2007.05.004 17604180

